# A comparative study of the bone contact to zirconium and titanium implants after 8 weeks of implantation in rabbit femoral condyles

**DOI:** 10.1007/s10266-017-0296-3

**Published:** 2017-02-13

**Authors:** Abdullah Aldosari AlFarraj, Anil Sukumaran, Mohammad D. Al Amri, AJA Bart Van Oirschot, John A. Jansen

**Affiliations:** 1 Department of Prosthetic Dental Science, College of Dentistry, King Saud University, Riyadh, Saudi Arabia; 2 Division of Periodontics, Department of PDS, College of Dentistry Prince Sattam Bin Abdulaziz University, PO Box 153, AIkharj, Riyadh, 11942 Saudi Arabia; 3 Department of Biomaterials, Radboud University Medical Center, Dentistry 309, PO Box 9101, Nijmegen, 6500 HB The Netherlands; 4 Dental Implant and Osseointegration Research Chair, (DIORC), College of Dentistry, King Saud University, Riyadh, Saudi Arabia

**Keywords:** Titanium, Zirconium, Calcium phosphate coating, Hydroxyapatite, Osseointegration, Rabbit model

## Abstract

Zirconium (Zr) has been found to have comparable characteristics to titanium with a favorable modulus of elasticity. In addition, the release of Zr-ions of a Zr implant is supposed to further increase the bone-to-implant response. Therefore, the objective of this study is to compare the bone contact to Zr and Ti implants in the femoral trabecular bone of rabbits. In addition, implants provided with a hydroxyapatite (HA) coating were included, as such a coating was proven before to enhance the secondary implant stability. A total of 32 implants consisting of 16 Zr (8 HA coated) and 16 Ti (8 HA coated) implants were installed in the femoral condyle of 16 rabbits. After 8 weeks of healing the femoral condyles including the implants were retrieved and studied histologically. The bone-to-implant contact (BIC) percentage was assessed and analyzed statistically. The BIC values of the uncoated Zr and Ti implants showed comparable BIC values (45.1 ± 14.8 vs. 45.5 ± 13.1). The BIC percentage was slightly higher for HA coated Zr and Ti implants (60.3 ± 17.1, 59.8 ± 16.4, respectively) compared to uncoated, but statistical testing indicated that this difference was not significant. It can be concluded that Zr and Ti implants show a comparable bone-implant contact after 8 weeks of implantation in the currently used rabbit model. In addition, the deposition of a sputtered HA coating on both Zr and Ti implants did not further improve their bone integration.

## Introduction

The primary and subsequent secondary stability are the major parameters for the clinical success of oral implants, which is depending on the biological as well as mechanical properties of the used implant material. An implant material has to be corrosion resistant, as characterized by the presence of a thin, passivating oxide layer, which repairs itself rapidly if damaged. Considering the required biomechanical characteristics, the modulus of elasticity of the implant material has to match the modulus of elasticity of bone, which will create a uniform distribution and an adequate reduction of the stresses at the implant-bone interface.

Currently, commercially pure titanium (Ti) is used extensively in the field of oral implantology because of its excellent mechanical strength, corrosion resistance, and good biocompatibility [1, 2]. These properties are attributable mainly to the formation of a stable titanium oxide layer on the surface [3]. The modulus of elasticity (E-modulus = Young’s modulus) of Ti is 116 GPa, which is favorable compared with the E-modulus of other materials as used for the manufacturing of implants (cobalt–chromium alloy = 200–220 GPa, stainless steel 316L = 200 GPa). Nevertheless, the E-modulus of Ti is still significantly higher than the E-modulus of bone, which ranges 0.5–30 GPa and is determined by the type and anatomical location of the bone tissue (trabecular vs. cortical, limbs vs. vertebral column vs. skull).

Zirconium (Zr) is a metal (in contrast to the ceramic material, zirconia), which is not widely used yet as implant material, but has chemical properties similar to those of Ti with no local or systemic toxic effects [4]. Although, Zr is also covered with a very stable and biologically inert oxide layer, the physical and chemical properties, such as oxidation rates, crystal structures, transport properties, water interactions, of the two metals Ti and Zr and their oxides differ quantitatively. Further, the E-modulus of Zr is 88 GPa and closer to the E-modulus of bone.

Bone is composed of an organic matrix (mainly collagen) and an inorganic component (mainly hydroxyapatite = HA). Calcium and phosphate compounds, in different configurations, make up for the major part of hydroxyapatite. However, the inorganic component of bone tissue contains also other inorganic composites, like zinc, strontium, copper, magnesium and fluoride [5]. It has been described that these inorganic trace elements do have osteogenic properties and can replace biological growth factors to influence bone development [6]. Cell culture studies demonstrated that the trace elements had an effect of the proliferation and differentiation of osteoblasts and the resorptive activity of osteoclasts. It was suggested that the bone biological performance of biomaterials can be modified by adding inorganic additives.

In view of the above mentioned, it has also been reported recently that ZrO2 and Zr-ions are able to promote the proliferation and differentiation of human osteoblasts in vitro [7, 8]. This effect is supposed to occur by up-regulation of BMP-2 expression and increased signaling. Therefore, we hypothesized that dental implants, manufactured of zirconium, do not only offer a biomechanical advantage but can also further increase the bone-implant response due to the release of ZrO2 as well as Zr-ions. Therefore, it can be hypothesized that the use of Zr, as implant material, will have a favorable effect on the implant-bone response.

Earlier studies showed already that surface modification of dental implants by the deposition of a synthetic HA coating on titanium can enhance the implant-bone response [9–12]. Hence, the objective of the present study is to analyze and compare the implant-bone response of Ti and Zr implants with and without HA coating. Therefore, Ti and Zr implants with and without HA coating were installed into the trabecular bone of the femoral condyle of rabbits and left in place for 8 weeks. Subsequently, bone-implant contact was assessed by light microscopic evaluation.

## Materials and methods

Sixteen healthy New Zealand White rabbits aged 6–9 months and weighing between 3.5 and 5 kg were used as experimental animals. All rabbits were housed separately in standard cages under laboratory conditions. The study was approved by the ethical committee of the College of Dentistry, King Saud University. The procedure was based on a well-established bilateral, rabbit, femoral condyle model [13].

### Implants

Thirty-two custom made implants (Machinefabriek Jansen BV, Valkenswaard, The Netherlands) were used for this study. Sixteen of them were made of commercially pure titanium (cpTi). The second group of 16 implants was made of medical grade Zr. The composition of the Zr is given in Table 1. Half of the implants from both groups were sputter coated with hydroxyapatite [14]. The study group consisted of four different types (8 implants in each group), i.e.: Ti, HA coated Ti, Zr and HA coated Zr implants.

**Table 1:** The composition of the zirconium

Type Zr 702	
Elements	Composition (%)
Zr + Hf	99.2
Hf maximum	4.5
Fe + Cr	≤0.2
Sn	–
H	0.005
N	0.025
C	0.05
Nb	–
O	0.16

**Fig. 1:**
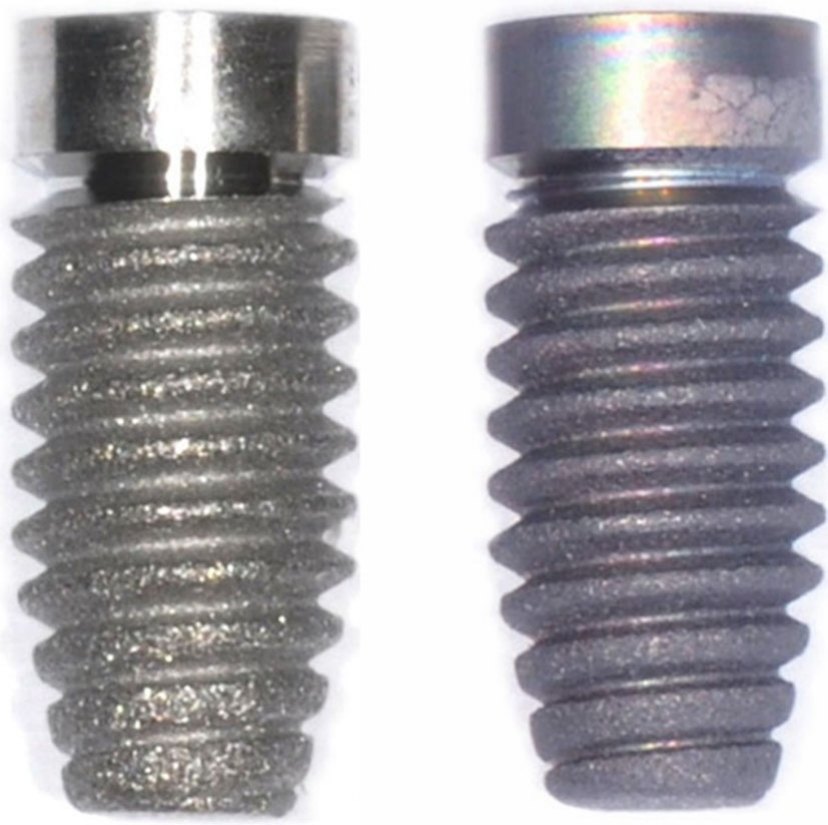
The type of implants used in the study (*Left* titanium, *Right* zirconium)

*CpTi and Zr implants* The cylindrical implants had the following dimensions: length of implant 8 mm, length of crestal part 1.5 mm, diameter at crestal side 3.5 mm, diameter at apex 2.6 mm, thread depth 0.5 mm, distance between threads 0.5 mm (Fig. 1). All implants were provided with a minimally rough surface by using a grit-blasting and acid etching procedure (Ra = 0.5 µm).

*HA coating procedure* HA coatings were deposited on the implants by using a commercially available RF sputter deposition system (Edwards ESM 100) [14]. The target material was composed of hydroxyapatite (Ca_5_(PO_4_)_5_OH) granules. The Ti and Zr implants were mounted on a rotating and water-cooled substrate holder. During deposition, the argon pressure was kept at 5 × 10^−3^ mbar and the sputter power was 400 W. After deposition, the coated implants were subjected to an additional infrared heat treatment (HT) for 30 s at 650 °C (Quad Ellipse Chamber, Model E4-10-P, Research Inc.). Before implantation, the composition and structure of the coatings were characterized by thin film X-ray diffraction (Philips, PW3710, Almelo, The Netherlands) and Fourier Transform Infrared spectroscopy.

Before surgical installation, all implants (as-received and coated) were autoclaved.

### Surgery

**Fig. 2:**
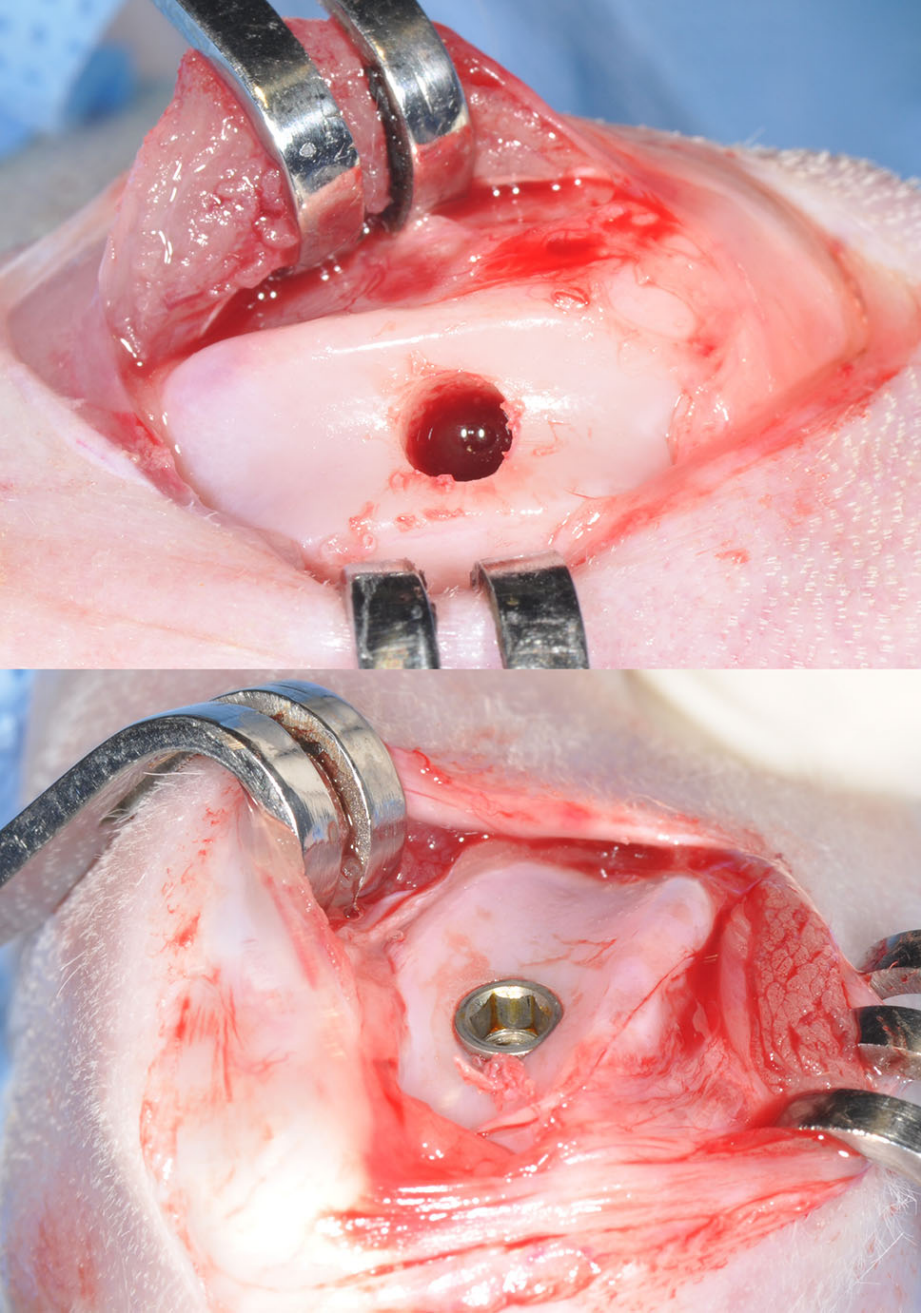
Pictures show the articular approach, as used for implant installation and final positioning of the implant

Surgery was performed using aseptic routines, and under general anesthesia by intramuscular injections of a combination dose of 35 mg/kg ketamine and a dose of 5 mg/kg xylazine. After anesthesia, the hind limbs were shaved, disinfected and isolated with drapes. Infiltration anesthesia was performed at the experimental sites. The left and right knee joints were exposed through a medial parapatellar longitudinal incision (Fig. 2). The capsule was incised and the medial femoral condyle was exposed after lateral dislocation of the patella. With the knee maximally flexed a hole was created through the articular cartilage into the subchondral bone on the weight-bearing surface of the femoral condyle using a dental drill (type, etc.). Drilling was performed at low rotational speed (600–800 rpm) using extensive external cooling with sterile saline solution. The implant bed was widened in gradient steps with a final drill diameter of 5 mm twist drill (Nobel Biocare AB, Goteborg, Sweden). The implants were installed with sufficient primary stability (≥20 Ncm) using a torque wrench (Astra Tech Implant System, Goteborg, Sweden) according a pre-determined randomized sequence, i.e. modified Latin square (Table 2).

**Table 2:** Randomization schedule

Rabbit number	Left condyle	Right condyle
1	Ti	TiHA
2	Zr	ZrHA
3	TiHA	Zr
4	ZrHA	Ti
5	Zr	ZrHA
6	Ti	TiHA
7	ZrHA	Ti
8	TiHA	Zr
9	Ti	TiHA
10	Zr	ZrHA
11	TiHA	Zr
12	ZrHA	Ti
13	Zr	ZrHA
14	Ti	TiHA
15	ZrHA	Ti
16	TiHA	Zr

*Ti* titanium, *TiHA* titanium coated with HA, *Zr* zirconium, *ZrHA* zirconium coated with HA

Finally, the surgical sites were closed in layers using resorbable sutures (Vicryl, 4–0) and animals were returned to their cages. Post-surgical pain was controlled by the administration Fynadyne^®^ (Fynadine, Schering Plough Animal Health Benelux, Utrecht, the Netherlands) intramuscularly. To reduce the post-operative infection risk Enrofloxacin 5–10 mg/kg (Baytril, Bayvet Division, Chemagro Ltd, Etobicoke, Ontario) (5–10 mg/kg) was administered. After 8 weeks of implantation, the animals were euthanized and the femoral condyles were harvested for histological preparation.

### Histology

After harvesting, all specimens were cleaned from attaching soft tissues. The femoral condyles including the implants were fixed in 10% formaldehyde. After fixation, the specimens were reduced in size and then dehydrated in increasing ethanol concentrations (70–100%). Finally, they were embedded (non-decalcified) in modified methyl-methacrylate (MMA) for 5 days (mixture of 300 ml MMA, 30 ml Dibutylphthalate, and 5 g 2,-2′azabisisobutyronitrile 98%). After polymerization in MMA, thin sections (10 μm) were cut in longitudinal direction to the axis of the implant using an inner circular saw microtome (Leica RM 1600, city, Germany). These sections were stained with methylene blue and basic fuchsin and were used for light microscopic assessment and histomorphometrical analysis.

### Histomorphometry

Histological evaluation was performed using the automated Zeiss Z1 Axio Imager microscope (Carl Zeiss Micro Imaging GmbH, Göttingen, Germany). Histomorphometry was performed using digital image analysis software (Leica^®^ Qwin Pro-image analysis, Leica Microsystems Imaging Solutions Ltd, Cambridge, UK). The quantitative parameter, as assessed, was the percentage of bone to implant contact (BIC %). Bone contact was analyzed along the total length of the implant; starting at the first coronal micro-thread up to the apex of the implant. BIC % was defined as the percentage of the implant surface in direct contact with bone without intervening fibrous tissue layers. Separate high resolution RGB (red, green, blue) microscopic images of the stained sections (10 × objective) containing the complete implants were acquired and stitched. After image binarization and subsequent thresholding the implant from bone, the length of the threaded implant surface was measured (contour of the implant surface was followed) and defined as the maximum possible bone-to-implant contact (BIC). The regions of agreement between the surface outline and the bone were measured as the real BIC parts. Finally, as a result, the BIC percentage was defined.

All measurements were performed for both sides of the implant on three at randomly selected histological sections per implant.

## Statistical analysis

All statistical analyses were performed with GraphPad^®^ Instat 3.05 software (GraphPad Software Inc, San Diego, CA, USA) using one-way analysis of variance (ANOVA) and t test. Tukey–Kramer Multiple Comparisons Test was used to analyze the groups. Differences were always considered significant at *P* values less than 0.05.

## Results

### Macroscopic evaluation

All animals survived the experimental period, stayed healthy and showed no sign of infection or discomfort. All implantations sites healed well and no swelling or redness was observed. Gross examination of the retrieved specimens indicated that all implants were in place without any sign of an inflammatory response. The articulating surface of the knee joint had a smooth appearance, but was not completely covered with cartilage. The implant could easily be recognized.

### Histological analysis

**Fig. 3:**
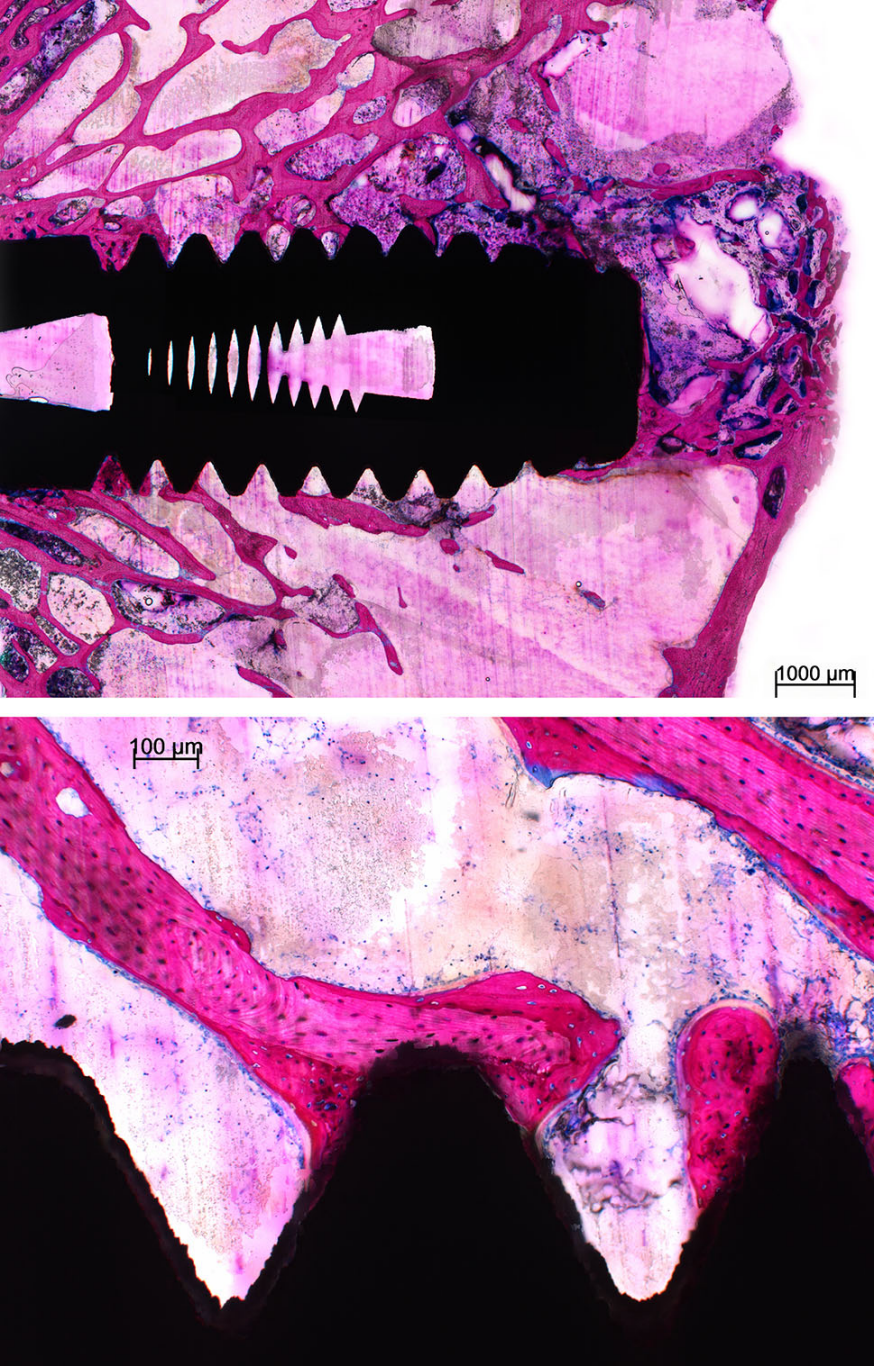
Light micrographs showing a Ti implant installed into the trabecular bone of the femoral condyle. The implant does not penetrate the growth plate. The bone trabeculae show a tentacle-like contact with the tip of the implant threads

**Fig. 4:**
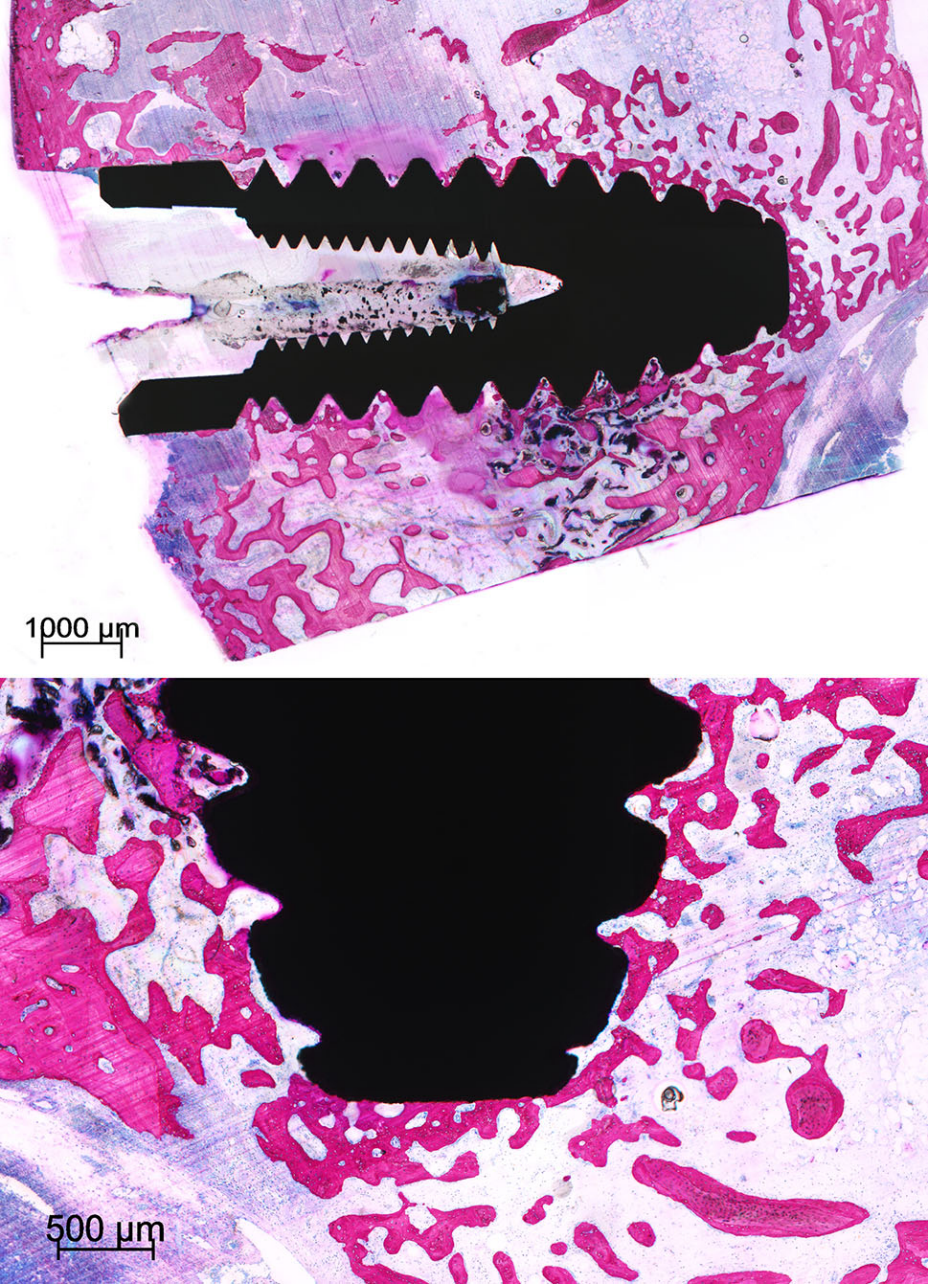
Histological section of a Ti-HA implant. At the crestal side only limited bone resorption is observed at one side of the implant. At the apex of the implant an abundance of newly deposited trabecular bone is visible. The bone is in close contact with the implant surface without the presence of an intervening fibrous tissue layer

**Fig. 5:**
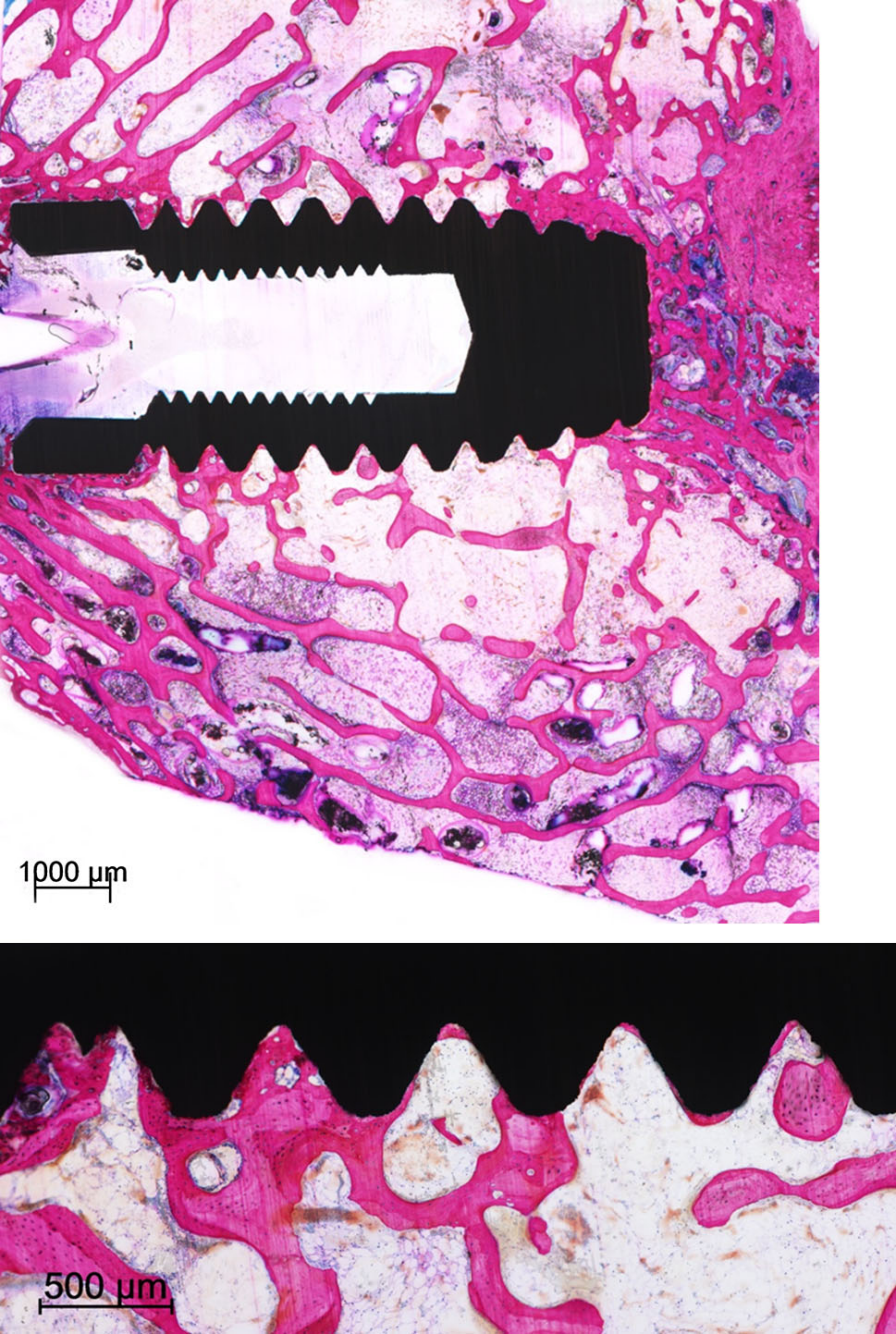
Light micrographs showing a Zr-HA implant. The implant is positioned into trabecular bone. The trabecular bone seems to be guided into the implant threads. No crestal resorption is observed

No gross differences in bone response were found between the various types of implants under light microscopy (Figs. 3, 4, 5). The implants were surrounded by trabecular bone, as characterized by the occurrence of bone trabeculae with interspersed areas of bone marrow (Figs. 3, 4, 5). The bone trabeculae showed direct contact with the implant surface. Frequently, this contact occurred at the tip of the implant thread. Subsequently, bone was observed to be guided into the implant thread and covered, as a thin layer, the implant surface. No inflammatory cells were seen in the bone-implant interface. The implant bed had completely remodeled and the original drill hole or loosened bone debris could occasionally only be recognized at the cortical side. At the apex of the implant, new bone formation had occurred and when the implant bed was somewhat oversized in length, the created space was always filled with bone tissue. At the cortical side, no or limited marginal bone resorption was observed and no overgrowth of cartilage had occurred (Figs. 4, 5).

### Histomorphometry

**Fig. 6:**
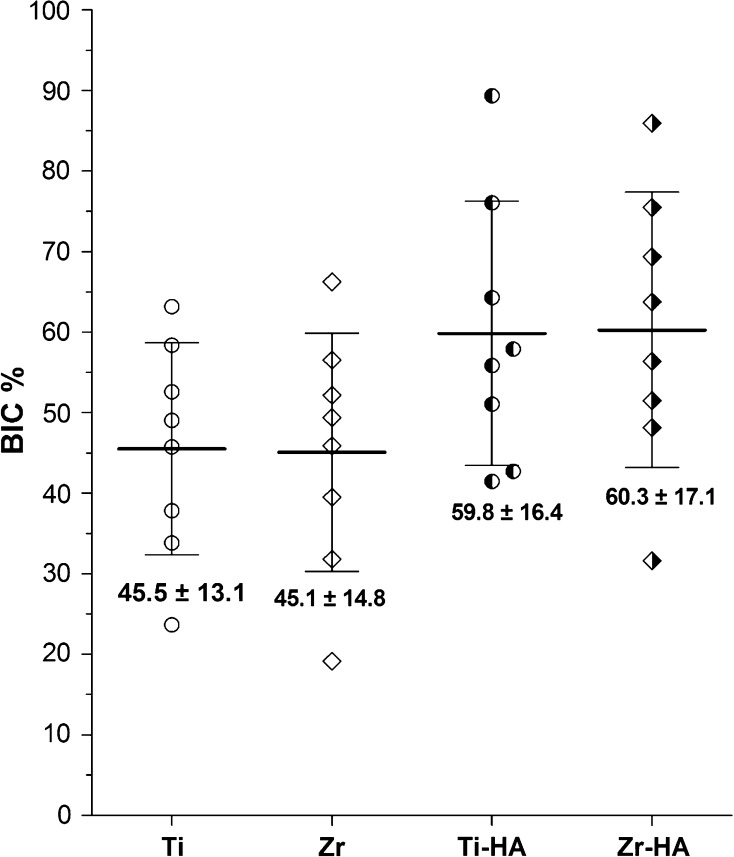
Bone-to-implant contact percentage (BIC %) in four types of implants used in the study (*Ti* titanium, *Zr* zirconium, *Ti-HA* titanium hydroxyapatite, *Zr-HA* zirconium hydroxyapatite). Graph is showing the distribution of the data

The BIC percentage of all four types of implant is depicted in Fig. 6. The BIC percentage of Zr and Ti implants showed comparable values (45.1 ± 14.8 and 45.5 ± 13.1, respectively). The HA coated Zr implants had a mean BIC percentage of 60.3 ± 17.1 compared to the HA-coated Ti implants with a mean BIC percentage of 59.8 ± 16.4. No statistical significance in BIC percentage existed between the various types of implants. Although, the BIC values seemed to be higher for HA coated Zr and Ti implants, the observed difference was not statistically significant. Further, it was noticed that the BIC measurements for the respective implant types showed a wide variation.

## Discussion

The surface composition of an implant plays an important role in determining the bonding between the implant and the newly formed bone tissue [15]. It determines the cellular and molecular response of osteoblasts and other cells that come in contact with the implant [10, 16]. Previous publications confirmed that the in vitro response of osteoblasts and osteoclasts was affected by inorganic trace elements [7, 8]. In contrast, this was not observed in our in vivo experimental animal study. Various explanations can be given for this finding, but firstly it has to be noticed that in the cell culture studies relatively large amounts of trace elements were added to the respective culture media. Under in vivo conditions, substantial ion release from the highly passive Zr material is depending on: (1) the occurrence of shear stresses at the implant-bone interface, which have to trigger the corrosion process, and (2) the pH found in the tissue surrounding the implant during the initial stages of wound healing [17, 18]. As implant movement in our experimental design is very limited due to the high initial stability of the implants, a very low ion release and a very low level of Zr-ions can be expected [19]. Also the ZrO_2_ film, covering the bulk Zr material, is highly adherent and prevents the release of corrosion products. In addition, a reduction of the pH of the implant bed will not contribute a lot to an increase of the corrosion rate. It can be supposed that the pH will already be restored within a few days after surgery to the physiological level due to the used atraumatic surgical procedure.

Our data indicated also that the deposition of an HA-coating on the Zr as well Ti implants, did not result in a statistically enhanced bone response. These results do not corroborate with earlier studies, where a similar HA-coating was found to accelerate initial stabilization of implants by enhancing bony ingrowth and stimulating osseous apposition to the implant surface [12, 20–24]. This discrepancy in bone response can be explained by the animal model used in this study and the implant design. Rabbits are known to show a faster skeletal change and bone turnover rate in comparison to large animal models, like goats, dogs and mini pigs [25]. The length of the bone remodeling cycle is 6 weeks in the rabbit compared with about 4 months in humans [26]. In majority of the previous studies, goats were used as experimental animal model and implants were left in place for 6–12 weeks. In these studies, the HA sputter-coated implants always showed an enhanced bone-to-implant contact compared with as-received titanium implants [9, 12, 13]. However, goats are known to show a prolonged bone healing response compared with small experimental animals. As HA-coatings are only able to affect the early bone response, it can be hypothesized that an implantation period of 8 weeks in a rabbit is too long to observe any difference in bone behavior. On the other hand, Hulshoff et al. [27] used rabbits. They installed HA sputter coated implants in the femoral condyle of rabbits and left them in place for 3, 6 and 9 weeks. Plasma-spray HA coated implants were used as reference material and the implant design was completely different. Implants had a cylindrical shape without the presence of screw-threads and were inserted press-fit into the implant bed. This involves that the implant diameter is exactly similar to the implant bed diameter. As a consequence, initial stability of these implants is less compared with the initial stability of a screw-type implant installed in an undersized implant bed. Initial stability affects the process of the secondary stability, which evidently can be overcome by using a bioactive HA-coating. This is also true for the studies, where rats were used and a beneficial effect of a sputtered HA-coating was observed. Also, these implants were not provided with screw-threads and were installed in a press-fit mode. As a consequence, the differences in experimental conditions do not allow a straight forward comparison of the various studies. In view of this, it has to be emphasized that researchers should be more focused on the selection of an appropriate animal model and implant design regarding their research question.

Finally, the histological and histomorphometrical analysis showed also that the hypothetically advantageous E-modulus of the Zr implants did not favor the initial bone response compared with Ti implants. An articular approach was used in the current study design to install the implants in the trabecular bone on the femoral condyle of the rabbits. This method has advantages over other approaches: (1) the implants are always completely surrounded by trabecular bone, and (2) the implants are loaded by the articulating tibial surface during movement of the rabbit, which mimicks the loading of dental implants during the initial healing phase. The procedure is simple and does not induce serious discomfort for the animals. The histological sections clearly showed that all implants were completely surrounded with trabecular bone without interfering with the growth plate [13]. The disadvantage of this installation approach is that loading condition of the implants is completely different compared with dental implants installed in the alveolar ridge of the maxilla or mandible. Therefore, it is difficult to make a final conclusion about the suggested effect of E-modulus of an implant material on bone behavior. It is recommended that additional studies are done in an animal model and with loading conditions that are more similar to the human clinical situation.

## Conclusion

On basis of the available data, we conclude that Zr and Ti show a comparable bone healing response after 8 weeks of implantation in the currently used rabbit model. In addition, the deposition of a sputtered HA coating on both Zr and Ti implants did not further improve their bone integration.
